# mGluR_5_ and GABA_A_ receptor‐specific parametric PET atlas construction—PET/MR data processing pipeline, validation, and application

**DOI:** 10.1002/hbm.25778

**Published:** 2022-01-25

**Authors:** Nicolas Kaulen, Ravichandran Rajkumar, Cláudia Régio Brambilla, Jörg Mauler, Shukti Ramkiran, Linda Orth, Hasan Sbaihat, Markus Lang, Christine Wyss, Elena Rota Kops, Jürgen Scheins, Bernd Neumaier, Johannes Ermert, Hans Herzog, Karl‐Joseph Langen, Christoph Lerche, N. Jon Shah, Tanja Veselinović, Irene Neuner

**Affiliations:** ^1^ Forschungszentrum Jülich Institute of Neuroscience and Medicine 4, INM‐4 Jülich Germany; ^2^ Department of Psychiatry, Psychotherapy and Psychosomatics RWTH Aachen University Aachen Germany; ^3^ JARA BRAIN Translational Medicine Aachen Germany; ^4^ Department of Medical Imaging Arab‐American University Palestine Jenin Palestine; ^5^ Forschungszentrum Jülich Institute of Neuroscience and Medicine 5, INM‐5 Jülich Germany; ^6^ Department for Psychiatry, Psychotherapy and Psychosomatics Social Psychiatry University Hospital of Psychiatry Zurich Zurich Switzerland; ^7^ Department of Nuclear Medicine RWTH Aachen University Aachen Germany; ^8^ Forschungszentrum Jülich Institute of Neuroscience and Medicine 11, INM‐11 Jülich Germany; ^9^ Department of Neurology RWTH Aachen University Aachen Germany

**Keywords:** GABA_A_, mGluR_5_, multimodal imaging, PET atlas construction

## Abstract

The glutamate and γ‐aminobutyric acid neuroreceptor subtypes mGluR_5_ and GABA_A_ are hypothesized to be involved in the development of a variety of psychiatric diseases. However, detailed information relating to their *in vivo* distribution is generally unavailable. Maps of such distributions could potentially aid clinical studies by providing a reference for the normal distribution of neuroreceptors and may also be useful as covariates in advanced functional magnetic resonance imaging (MR) studies. In this study, we propose a comprehensive processing pipeline for the construction of standard space, *in vivo* distributions of non‐displaceable binding potential (*BP*_ND_), and total distribution volume (*V*_T_) based on simultaneously acquired bolus‐infusion positron emission tomography (PET) and MR data. The pipeline was applied to [^11^C]ABP688‐PET/MR (13 healthy male non‐smokers, 26.6 ± 7.0 years) and [^11^C]Flumazenil‐PET/MR (10 healthy males, 25.8 ± 3.0 years) data. Activity concentration templates, as well as *V*_T_ and *BP*_ND_ atlases of mGluR_5_ and GABA_A_, were generated from these data. The maps were validated by assessing the percent error δ from warped space to native space in a selection of brain regions. We verified that the average δ_ABP_ = 3.0 ± 1.0% and δ_FMZ_ = 3.8 ± 1.4% were lower than the expected variabilities σ of the tracers (σ_ABP_ = 4.0%–16.0%, σ_FMZ_ = 3.9%–9.5%). An evaluation of PET‐to‐PET registrations based on the new maps showed higher registration accuracy compared to registrations based on the commonly used [^15^O]H_2_O‐template distributed with SPM12. Thus, we conclude that the resulting maps can be used for further research and the proposed pipeline is a viable tool for the construction of standardized PET data distributions.

## Graphical abstract

In this study, we propose a comprehensive processing pipeline for the construction of standard space, *in vivo* distributions of non‐displaceable binding potential (*BP*_ND_), and total distribution volume (*V*_T_) based on simultaneously acquired bolus‐infusion positron emission tomography (PET) and magnetic resonance imaging (MR) data. The pipeline was applied to [^11^C]ABP688‐PET/MR (13 healthy male non‐smokers, 26.6 ± 7.0 years) and [^11^C]Flumazenil‐PET/MR (10 healthy males, 25.8 ± 3.0 years) data. Activity concentration templates, as well as *V*_T_ and *BP*_ND_ atlases of mGluR5 and GABAA, were generated from these data.

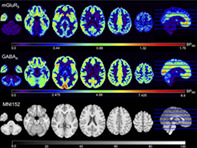

## 1 INTRODUCTION

The advantages of simultaneously acquired positron emission tomography (PET) and magnetic resonance imaging (MR) data have been extensively reported in numerous previous studies (Herzog et al., 2011; Herzog, 2012; Catana, Drzezga, Heiss, & Rosen, 2012; Torigian et al., 2013). Simultaneous PET/MR brain imaging enables the combination of *in vivo* data relating to neuroreceptor systems (obtained from PET) with anatomical and structural information acquired under exactly the same conditions using multiparametric MR (Sander, Hansen, & Wey, 2020). This application provides a perfect basis for the construction of specific neuroreceptor distribution maps. Brain atlases showing standard distributions of the most important neuroreceptors in the healthy brain may aid the study of the molecular mechanisms underlying psychiatric conditions. Furthermore, such maps may also be useful as covariates in functional MR (fMRI) studies. Indeed, an association between the fMRI signal, the relative receptor occupancy, and the level of neurotransmitter (concretely dopamine) has been demonstrated previously (Mandeville et al., 2013). Thus, consideration of receptor availability provides additional information for more advanced analyses of fMRI studies.

In this study, we aimed to construct atlases showing parametric total volume of distribution (*V*_T_) and non‐displaceable binding potential (*BP*_ND_), in conjunction with normalized activity concentration templates, based on bolus‐infusion PET and simultaneously acquired MR data. For this purpose, we designed a coherent PET/MR neuroimaging processing pipeline in NiPype (Gorgolewski et al., 2011). Based on data from healthy subjects, the pipeline was then used to establish *in vivo* maps of *V*_T_ and *BP*_ND_ for use as a reference in advanced studies of psychiatric and neurologic diseases. The pipeline was further used in the creation of [^11^C]ABP and [^11^C]FMZ activity concentration templates to provide tracer‐specific target templates for PET‐to‐PET registration.

To test the applicability of our method, two radioligands for which no established receptor atlases or activity concentration templates were available were selected: [^11^C]ABP688 (3‐(6‐methyl‐pyridine‐2‐ylethynyl)‐cyclohex‐2‐enone‐O‐[^11^C]methyloxime) and [^11^C]Flumazenil.

[^11^C]ABP688 is a recently developed radioligand that binds to the allosteric site of the metabotropic glutamate receptor subtype 5 (mGluR_5_; Ametamey et al., 2007). Previous investigations have shown sufficient test–retest reliability for this radiotracer (Smart et al., 2018) and have demonstrated its ability to detect physiological changes in endogenous glutamate levels (DeLorenzo et al., 2011). Glutamate is the main excitatory neurotransmitter in the brain (Meldrum, 2000), and disturbances in the glutamatergic system are hypothesized to be involved in the development of numerous psychiatric and neurological diseases, including Parkinson's disease, depression, anxiety, and schizophrenia (Niswender & Conn, 2010).

[^11^C]Flumazenil is a well‐established and widely used radiotracer that binds to the benzodiazepine binding site of the γ‐aminobutyric acid class A (GABA_A_) receptors (Odano et al., 2009). GABA is the main inhibitory neurotransmitter in the brain (Petroff, 2002), and disturbances in GABAergic neurotransmission is associated with several psychiatric diseases such as major depressive disorder, schizophrenia, and bipolar disorder (Chiapponi, Piras, Piras, Caltagirone, & Spalletta, 2016), as well as anxiety disorders, epilepsy, and insomnia (Möhler, 2006).

As the two selected ligands (in the following abbreviated as [^11^C]FMZ and [^11^C]ABP) are suitable for the investigation of the fundamental inhibitory and excitatory neurotransmitters involved in several psychiatric and neurologic diseases, the generation of atlases for the corresponding receptors is of high scientific importance. In this work, we demonstrate the validity of our pipeline for the creation of *BP*_ND_ and *V*_T_ maps and evaluate whether the resulting atlases reflect accurate parametric values by performing region‐of‐interest (ROI) analyses in a native space and a template space.

We also investigate the effect of omitting the parameter estimation step from the proposed pipeline on the generation of normalized activity concentration templates for use in direct PET‐to‐PET registration, as this would be particularly beneficial for PET imaging applications that focus on specific neurotransmitter systems. Direct PET‐to‐PET normalization is usually necessary for conducting group‐wise evaluation when PET data are acquired without an MR image. Although hybrid PET/MR systems are gaining relevance in research and have significant diagnostic advantages over the more commonly used PET‐CT (computed tomography; Von Schulthess & Schlemmer, 2009; Zaidi, Mawlawi, & Orton, 2007), they remain rare in clinical applications (Ehman et al., 2017). If no structural MR image is available, the registration of the PET image onto a template is usually conducted using the standardized perfusion data represented by the [^15^O]H_2_O PET template, which is distributed with the MATLAB‐based neuroscientific image analysis package Statistical Parametric Mapping (SPM12; Penny, Friston, Ashburner, Kiebel, & Nichols, 2011). Apart from the [^15^O]H_2_O template, a 2‐[^18^F]fluoro‐2‐deoxy‐D‐glucose template was constructed and confirmed as giving more accurate registration in diagnostic applications (Della Rosa et al., 2014). However, both of the aforementioned templates primarily show the distribution of gray matter, which barely coincides with the distribution of neuroreceptors. Therefore, the use of specific templates is more beneficial, as already demonstrated for two carbonyl‐^11^C‐labeled tracers—[^11^C]WAY‐100635 and [^11^C]Raclopride (Meyer, Gunn, Myers, & Grasby, 1999). Furthermore, a dynamic 4D [^11^C]Raclopride template has been constructed based on PET data acquired using a high‐resolution research tomography (Bieth, Lombaert, Reader, & Siddiqi, 2013). The usefulness of these types of registration templates has thus been demonstrated for the group‐wise evaluations of PET datasets (Della Rosa et al., 2014; Meyer et al., 1999). In this work, registration results from the newly created templates for [^11^C]FMZ and [^11^C]ABP were used as a comparison with the results obtained with the [^15^O]H_2_O template as a registration target.

## 2 METHODS

### 2.1 Subject selection and data acquisition

Twenty‐three subsets of data from two previous studies conducted in our institute using a hybrid BrainPET/MR scanner (Herzog et al., 2011) were finally considered for the construction of the novel atlases, and a subset from a larger study investigating the role of the mGluR_5_ in schizophrenia was used for the generation of the [^11^C]ABP maps. At the time of data analysis, 15 healthy male non‐smokers could be considered for further processing. Another subset of data from this study was recently analyzed and published (Régio Brambilla et al., 2020). The [^11^C]FMZ data originate from a study of 20 healthy male participants, which aimed to optimize the bolus‐infusion scheme for the [^11^C]FMZ acquisitions (Mauler et al., 2020). Both studies, including the presented analysis, were approved by the Ethics Committee of the Medical Faculty at the RWTH Aachen University and the German Federal Office for Radiation Protection (Bundesamt für Strahlenschutz). Written informed consent was obtained from all participants before the measurement.

The radiosynthesis of [^11^C]ABP and [^11^C]FMZ was performed according to literature methods (Canales‐Candela, Riss, & Aigbirhio, 2012; Elmenhorst et al., 2016). In both studies, the PET tracer was injected as a bolus plus constant infusion (B/I). Information about the mean injected activity, mean age of the participants, bolus fraction (Kbol), and the acquisition time for both studies is given in Table 1. The simultaneous PET/MR data acquisition protocol involved the concurrent acquisition of PET data in list‐mode with structural and functional MR data acquisition. Structural MR data were acquired immediately after the bolus injection, before the tracer reached the equilibrium state. Once the tracer was expected to have reached the steady‐state, a “resting state – task – resting state” paradigm was applied as described in Neuner et al. (2018). In the case of [^11^C]FMZ, the considered list‐mode PET data were framed at 20 × 5 min, and 20 × 2 min framing was used to reconstruct the first 40 min of the [^11^C]ABP data. The framed data were reconstructed into a 256 × 256 × 153 image matrix with a 1.25 × 1.25 × 1.25 mm isometric voxel size, using 3D ordinary Poisson‐ordered subset expectation maximization (3D OP‐OSEM) with 32 iterations and two subsets (Zhang et al., 2014). To eliminate any effects, the tasks in the measurement protocols may have had on the data, only frames up to the first task phase were considered for the purposes of this work. Thus, it was not necessary to reconstruct frames for the entire duration of the measurements. This is further explained in Section 2.2.2. During reconstruction, attenuation correction was applied based on an initially acquired MR image. Details relating to this template‐based attenuation correction method are discussed elsewhere (Kops & Herzog, 2008). Additionally, corrections for dead time, decay, random coincidences (variance reduction of randoms), and scatter were applied.

**TABLE 1:** Additional information regarding the groups of volunteers as well as the respective acquisition time (A_T_), administered dose (A_D_), and bolus fraction K_bol_ in the two considered studies

Study	*N*	Age (years)	A_D_ (MBq)	K_Bol_ (min)	A_T_ (min)	Gender	Non‐smokers
[^11^C]ABP	13	25.8 ± 3.0	410.0 ± 19.4	46.2	120	Male only	13/13
[^11^C]FMZ	10	26.6 ± 7.0	426.4 ± 61.2	61.8	65	Male only	N.A.

Abbreviation: N.A., not available.

The T_1_‐weighted, structural MR images were acquired with a magnetically prepared rapidly acquired gradient echo (MPRAGE) sequence in 176 sagittal slices of 1 mm thickness with the following MR parameters: repetition time (TR) = 2,000 ms, echo time (TE) = 3.03 ms, flip angle α = 9°, GRAPPA factor = 2.

### 2.2 Data preparation

The ECAT7 PET files and DICOM MR files were converted into the common NifTi file format using dcm2niix (Li, Morgan, Ashburner, Smith, & Rorden, 2016). This file format is readable with all of the relevant software that tools that were used in the pipeline. Two preliminary steps were applied: first, a brain extraction step was performed to increase the accuracy of the nonlinear registration step. Second, the PET files underwent a time‐activity curve (TAC) analysis to ensure that the equilibrium condition for accurate estimation of *BP*_ND_ and *V*_T_ was met.

#### 2.2.1 Brain extraction

**FIGURE 1:**
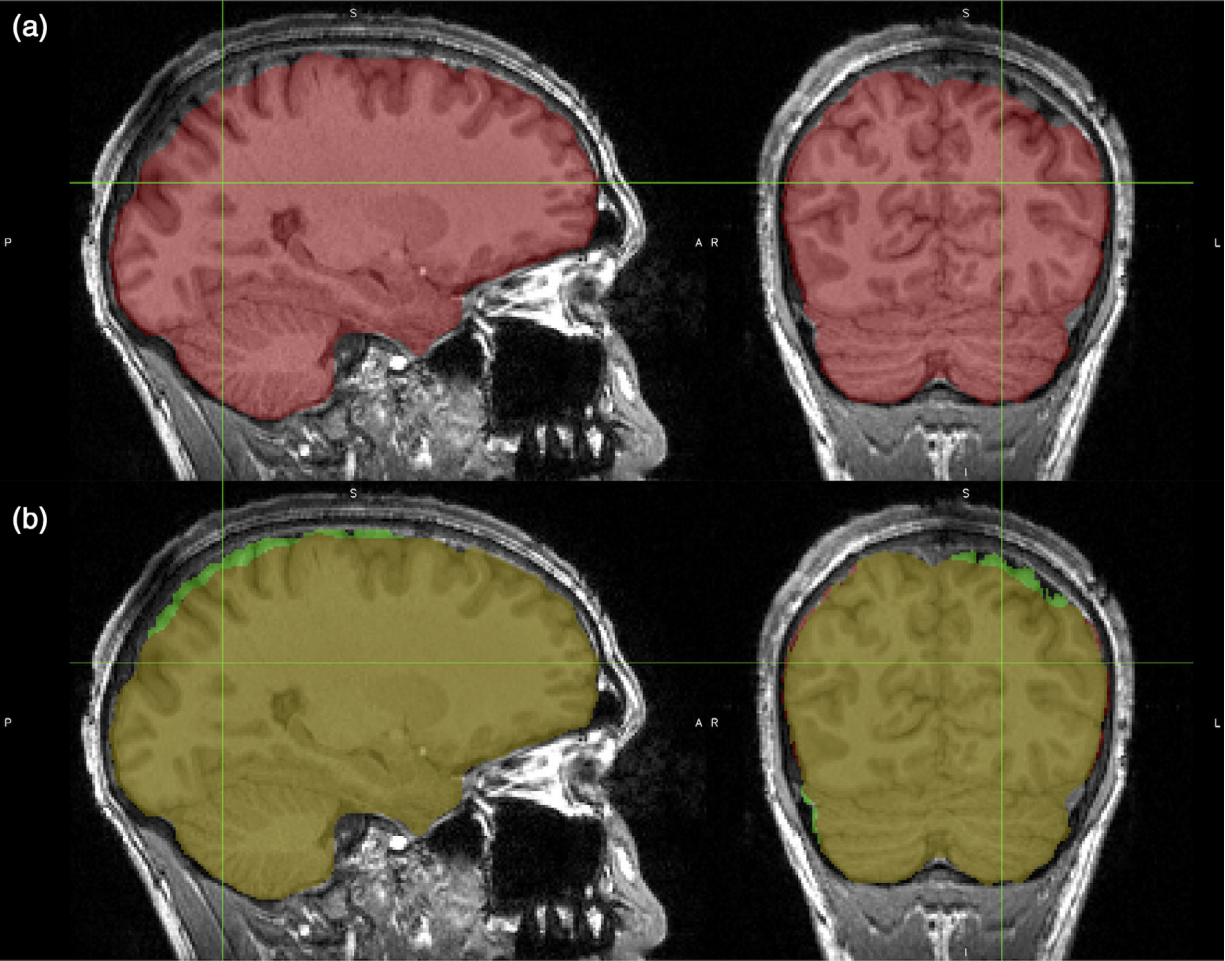
An example illustrating the manual correction of the brain masks. (a) The original mask output of the ANTs brain extraction script—red overlayed onto the structural MR scan. (b) The corrected mask in green overlayed on top of the original mask and the MR scan. The cursor is drawn in green and is located at (68, 54, 165)

In order to achieve maximal registration accuracy with the advanced normalization tools (ANTs) method (Avants et al., 2011), the use of skull‐stripped T_1_‐weighted images is recommended to estimate the optimal registration into template space (Pustina & Cook, 2017). Several skull‐stripping methods are established in neuroimaging. However, none of the tools tested in the course of this work (FSL BET, ANTs Brain Extraction, SPM NewSegment) were able to consistently output accurate brain extractions when using one fixed set of parameters. Thus, instead of individually optimizing the parameters to reach the needed performance of the scripts, the binary brain masks produced by the ANTs brain extraction shell script were each edited manually in the FSL image viewer FSLeyes (McCarthy, 2020) to ensure a minimal amount of missing or non‐brain tissue voxels. An example of manual correction for one [^11^C]FMZ subject is shown in Figure 1.

#### 2.2.2 TAC evaluation

For the estimation of *BP*_ND_ and *V*_T_ in the later stages of the pipeline, the equilibrium of the tracer in plasma and tissue must be already established. To ensure that a constant equilibrium at the time of interest was reached, TACs were plotted and analyzed prior to the pipeline processing. In both datasets, the start of the equilibrium was expected to occur approximately 30 min after injection (Neuner et al., 2018; Régio Brambilla et al., 2020). The TACs in the whole gray matter (GM) and the respective reference regions were normalized to the mean activity in GM during the expected equilibrium phase. Previous studies have shown that the GM of the cerebellum is a suitable reference region for [^11^C]ABP (Akkus et al., 2013; Régio Brambilla et al., 2020; Smart et al., 2018). The definition of the cerebellar GM in the Neuromorphometrics brain atlas included in SPM12 was used for *BP*_ND_ calculation in the ABP subjects. The data used to create this atlas originated from the OASIS project (www.oasis-brains.org), and the labels were provided by Neuromorphometrics, Inc., under academic subscription (www.neuromorphometrics.com).

In the case of [^11^C]FMZ, the pons is a brain region with a negligible amount of GABA_A_ receptors (Odano et al., 2009). Here, a manually drawn mask was created in FSLeyes, guided by the use of the MNI space template referred to in Section 2.3.2. The fuzzy edges of this mask were reduced by eroding it and then dilating it using the same spherical kernel for both operations.

Only scans that contained frames with a deviation below 10% of normalized activity concentration during the time of the first resting‐state phase were accepted for atlas construction. The TACs of the subjects finally considered, including the respective selected frames, are given in the Supporting Information Material.

A further purpose of the TAC evaluation was to find the frames in which the equilibrium condition was already established and coincided with the first resting‐state phase of the protocol mentioned in Section 2.1. Frames were accepted if they covered more than 50% of resting‐state acquisition. This way, any influence of the following tasks on the PET data could be ruled out. During resting‐state acquisition, the subjects were instructed to close their eyes and to stay calm without thinking about anything specific.

Following initial visual inspection, TAC evaluation, and motion correction, two subjects of the [^11^C]ABP study, and 10 subjects of the [^11^C]FMZ study were excluded from further consideration leaving a total of 10 subjects from the [^11^C]FMZ study and 13 healthy male non‐smokers from the [^11^C]ABP dataset.

### 2.3 NiPype image processing pipeline

The data for the construction of the PET atlases were pre‐processed using one coherent pipeline of processing steps that were assembled in NiPype (Gorgolewski et al., 2011)—a Python package comprising a variety of neuroimaging software wrapped into Python interfaces so that they can be connected in a single programming language. The use of NiPype enables multi‐step processing of entire sets of neuroimaging data. In addition, the processing approaches can be simply adapted to individual needs. The relevant NiPype dependencies for this work are Statistical Parametric Mapping (SPM12; Penny et al., 2011), ANTs (Avants et al., 2011), FMRIBs Software Library (FSL; Jenkinson, Beckmann, Behrens, Woolrich, & Smith, 2012), and PETPVC (Thomas et al., 2016). It should be noted here that a set of NiPype image processing workflows, named Pypes (Savio, Schutte, Graña, & Yakushev, 2017), is already publicly available. This collection of workflows also comprises a workflow for the MR‐based registration of PET images into a standardized space. The available workflows in Pypes, however, do not use implementations for PET parameter estimation, such as binding potential, or volume of distribution, to draw conclusions based on quantitative data. The performance of parameter estimation in transformed spaces is not recommended as it could potentially bias the results. Thus, one cannot use the outputs from the Pypes workflows as a basis for parameter estimation. In the approach proposed here, emphasis was placed on an optimized selection of pre‐processing steps to minimize error sources. This selection will be described in detail in the following paragraphs.

**FIGURE 2:**
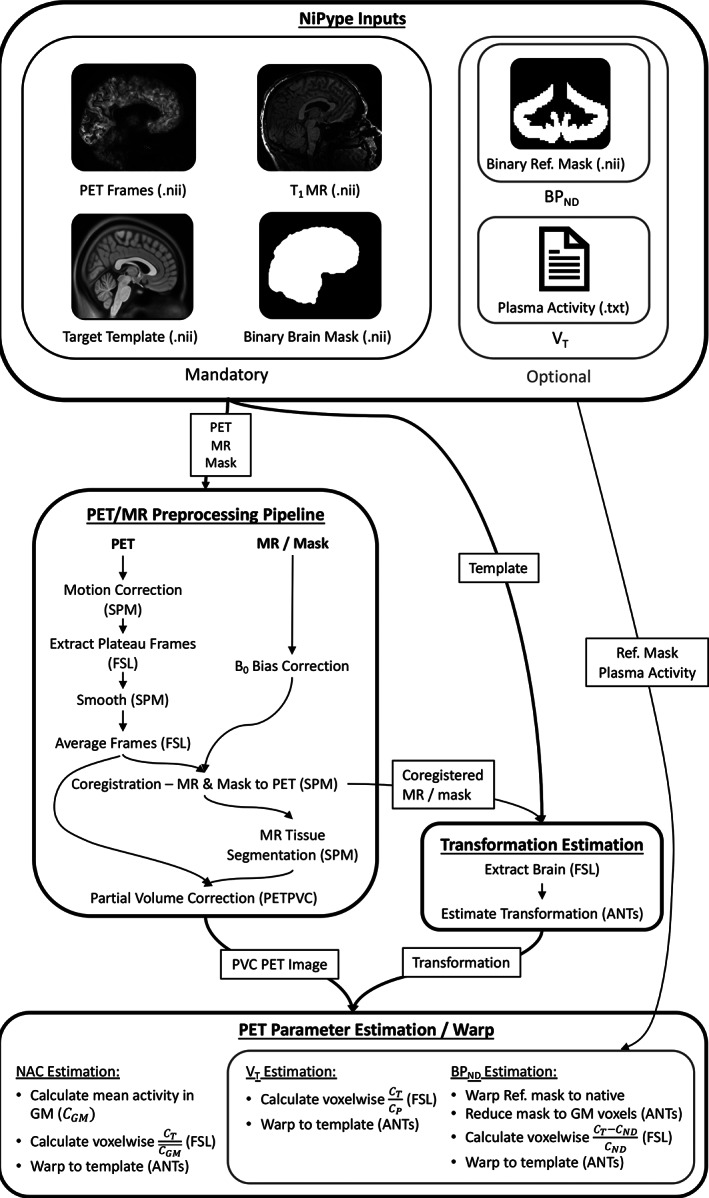
A graphical representation of the constructed NiPype preprocessing pipeline. ANTs, Advanced Normalization Tools; FSL, FMRIB's Software Library; SPM, Statistical Parametric Mapping

The image processing pipeline consists of three major parts: (a) MR and PET data pre‐processing; (b) estimation of the transformation into a standardized space and; (c) PET parameter calculation. A detailed graph illustration of the implemented pipeline is given in Figure 2.

To be able to apply the pipeline, the user is required to install the software requirements mentioned above and must simply provide the following.

1. The raw structural MR scans in addition to the respective binary brain masks.
2. The reconstructed and TAC evaluated PET data.
3. A target template file (i.e., in MNI Space).
4. A list of tuples, defining the frames of interest for each subject (starting frame no., no. of frames).
5. (Optional) For *V*_T_ estimation: metabolite corrected mean venous blood plasma activity at the time of interest as a text file.
6. (Optional) For *BP*_ND_ estimation: a binary NifTi file masking the reference region in template space.

Currently, the pipeline expects all neuroimaging data in NifTi format. Additionally, the FWHM of a Gaussian kernel for PET smoothing can be set, and there is a choice of whether or not to apply partial volume correction (PVC). With these inputs given, the processing steps outlined in the following sections were run fully automatically. For the purposes of this work, the FWHM is set to 2.5 mm and PVC is included.

#### 2.3.1 MR and PET data pre‐processing

Raw PET scans were initially motion corrected using the SPM12 routine “Realign.” The pipeline is set to apply motion correction with respect to the first frame. The frames of interest were extracted from the motion‐corrected frames according to the input tuple, smoothed (SPM; Gaussian kernel sized 2.5 mm FWHM), and averaged (FSL). After the bias‐correction of the raw structural MR scans using the ANTs N4 bias correction method, with the option to normalize the intensity range set to true (Tustison et al., 2010), the MR image and the input brain mask were co‐registered to the previously generated average PET frame by applying the SPM12 co‐registration method. The last step of this pipeline workflow is PVC. Therefore, the co‐registered MR images were segmented using SPM12 NewSegment, and the resulting tissue probability maps were given to an implementation of the RBV + Labbé approach within the PETPVC package.

#### 2.3.2 Transformation estimation

From the previous steps, the bias‐corrected and co‐registered MR images and brain masks were taken as input to the estimation of the transformations into the standard space. The masks were used to extract the brain segments from the whole‐head MR scan. The extracted brain images were then given as input to the ANTs registration method, which was set to calculate optimal transformations in a rigid, then an affine, and lastly a nonlinear symmetric normalization (SyN) step to match each voxel to the corresponding voxel in the template image. The ICBM152 2009c nonlinear asymmetric, 1 × 1 × 1 mm resolution brain template, available on the website of the McConnel Brain Imaging Center, Montreal Neurological Institute of the McGill University Montreal (Fonov, Evans, McKinstry, Almli, & Collins, 2009) was chosen as the registration target for this work. Unless explicitly stated, MNI space refers to the above‐mentioned MNI template space in the following work. The resulting transformation into MNI space (forward transform) and from MNI space back into subject space (inverse transform) were output from this pipeline segment for later purposes.

#### 2.3.3 PET parameter calculation

The advantage of the applied B/I infusion protocol is that it enables the estimation of *BP*_ND_ and *V*_T_ using a simple ratio method. The B/I acquisition protocol has been previously validated (Carson, 2000) and optimized for [^11^C]ABP (Burger et al., 2010) and [^11^C]FMZ (Mauler et al., 2020). Metabolite correction of venous blood plasma was performed following the method described in Mauler et al. (2020). At true equilibrium, it is possible to calculate *V*_T_ and *BP*_ND_ according to Equations (1) (Carson et al., 1993) and (2) using the activity concentration in tissue *C*_*T*_, plasma *C*_*P*_, and the non‐displaceable tissue concentration *C*_ND_

(1) $$V_{T}=\frac{C_{T}}{C_{p}},$$

(2) $$\;{\mathrm{BP}}_{\mathrm{ND}}=\frac{C_{T}-C_{\mathrm{ND}}}{C_{\mathrm{ND}}}.$$

The estimations of *BP*_ND_ and *V*_T_ were performed in subject space before the transformation into MNI space was applied. For *BP*_ND_ calculation, the MNI space reference region was warped into subject space using the inverse transform. For the estimation of *BP*_ND_ in the [^11^C]ABP subjects, the warped mask was multiplied with the individual SPM12 GM segment to exclude voxels unlikely to contain GM. Due to the interpolation steps during the transformation, voxel values at the border of the mask were changed so that the resulting mask was no longer binary. To calculate the mean value (*BP*_ND_, *V*_T_, or normalized activity concentration) fslstats was applied. By default, fslstats thresholds and binarizes the input mask at 0.5 and then calculates the mean value from all non‐zero voxels. This threshold is helpful to avoid the inclusion of these bordering voxels into the analysis, as well as for restricting the masks to voxels with a high probability of being GM. The output was then used to calculate *BP*_ND_ after (2). The same was performed for *V*_T_ following (1).

### 2.4 Pipeline outputs and template construction

To aid quality control, the pipeline was set to output several intermediate results: motion correction parameters, bias corrected and co‐registered MR, resulting tissue segments, mean PET frames of interest). In addition, the forward and inverse transforms estimated during the pipeline execution were also output, as well as the normalized activity concentration and parametric versions of the PET images in native and template space.

The templates were thus constructed by averaging the resulting files in template space. The *BP*_ND_ and *V_T_* versions of the templates were masked to remove irrelevant voxels. This was achieved by using a dilated version of the binary brain mask that is distributed in addition to the MNI template used for this work. The dilation kernel was set to a spherical kernel with a radius of 2.5 mm to ensure that no relevant information in the templates was cut off by the mask. The activity concentration templates were left unmasked.

## 3 VALIDATION

### 3.1 Warp effect assessment

Before being able to use the constructed templates for applications and analyses, a validation step was performed to verify the accuracy of the warped images. Assessment of the warp effect is important because nonlinear transformations introduce local and global volumetric changes as well as artifacts due to interpolation operations. Therefore, the error between values in the native image and the warped version needed to be estimated.

For the assessment, laterally separated ROIs, as defined in the Harvard Oxford (HO) atlas (Desikan et al., 2006), distributed with the functional connectivity toolbox, CONN (Whitfield‐Gabrieli & Nieto‐Castanon, 2012) were used. The cerebellar regions of this atlas were not considered in the following because their overlap with the used MNI template was poor. The analysis was applied using several intermediate results of the processing pipeline described above. The forward transformation of the nonlinear transformation into MNI space was applied to the native GM segment of each subject. The respective inverse transformation was used to transform the ROI mask from the atlas into native space. The native and warped space GM distribution maps given by SPM12 were multiplied with the native and warped space ROIs to restrict the ROIs to a close estimate of the true GM. With this operation, the previously binary ROIs were converted into probabilistic masks. For both the native space and the warped space, the average values in each region were calculated as weighted averages using the probabilistic values of the masks as weights. The percent error δ from the warped space value (*BP*_ND,*w*_) to the native space value (*BP*_ND,*n*_) was calculated for all regions in each subject according to Equation (3)

(3) $$\delta =\left|\frac{{\mathrm{BP}}_{\mathrm{ND},w}-{\mathrm{BP}}_{\mathrm{ND},n}}{{\mathrm{BP}}_{\mathrm{ND},n}}\right|\times 100.$$

### 3.2 PET‐to‐PET registration

In contrast to the parametric PET atlases, the warp effect is negligible for the construction of activity concentration templates since most current registration routines estimate the transformation based on relative voxel intensities using metrics such as normalized mutual information and cross‐correlation. Thus, relative changes in the image are more relevant than accurate absolute values.

As stated in Section 1, many users rely on the [^15^O]H_2_O template that is distributed with SPM12 to perform direct PET‐to‐PET registration due to the lack of standardized templates for most tracers. However, the [^15^O]H_2_O PET template in question was generated based on the ICBM 152 linear space (J. C. Mazziotta, Toga, Evans, Fox, & Lancaster, 1995; J. Mazziotta et al., 2001a, 2001b) according to the spm_templates.man file which contains Supporting Information Material about the available templates within the SPM12 package. Following visual inspection, noticeable differences between the [^15^O]H_2_O template‐space and the MNI template version that was used for registration purposes in this work were observed. Differences between the different iterations of the MNI templates are known. A comprehensive overview of the different versions is given in Lead‐DBS (n.d.).

Thus, PET‐to‐PET registrations based on the [^15^O]H_2_O template cannot be expected to be well aligned to the most recent MNI space. Therefore, in order to demonstrate the advantage of the newly constructed PET templates, we chose to compare [^15^O]H_2_O‐based registrations with registrations based on the current MNI152 space from 2009. This was achieved by considering two further selections of subjects from the same respective studies. The selected test files were unrelated to the target templates. Four healthy male subjects from the [^11^C]FMZ study (age = 25.8 ± 3 years, AD = 404.8 ± 5.6 MBq) and five male non‐smoker schizophrenia patients from the [^11^C]ABP study (age = 28 ± 6 years, AD = 497.8 ± 34.0 MBq) were considered for the registration tests. The non‐smoker schizophrenia patients were chosen due to a lack of healthy non‐smoker subjects that were not already used for template construction. However, a previous investigation found no significant differences in receptor availabilities of matched healthy controls in these patients (Régio Brambilla et al., 2020), so adequate image similarity for registration purposes was assumed.

Testing was carried out as follows: First, the considered data were preprocessed using the previously described pipeline. The resulting MR‐based registrations were considered as ground truth. Second, direct PET‐to‐PET registrations using the registration routines of SPM12 Old Normalize (SPM_ON_), as well as the quick tool antsRegistrationSyNQuick (ANTs_SQ_) of ANTs, were used to estimate PET‐based registration towards the [^15^O]H_2_O template and the two tracer‐specific templates. Two registration methods were used to investigate whether the outcome depends on the registration routine applied. SPM_ON_ was applied using default settings, and ANTs was set to perform one rigid, one affine, and one SyN registration stage and otherwise default settings. For the registration to the [^15^O]H_2_O perfusion template, the use of early uptake data is recommended to approximate a perfusion pattern (PMOD Technologies GmbH, 2021). Thus, the estimation of the registrations towards the [^15^O]H_2_O template was based on the first 5‐min frame of the [^11^C]FMZ subjects and an average of the first three 2‐min frames of the [^11^C]ABP subjects. The resulting transformations were then applied to the respective steady‐state frames of each subject. For the registrations to the tracer‐specific templates, the steady‐state frames can be used directly for transformation estimation. Subsequently, the similarity between the PET‐based and the MR‐based registration was measured in terms of the following image similarity metrics: the ANTs implementations of mutual information (MI), local cross‐correlation (*r* = 4 voxels, CC), and voxel‐wise mean squared error (MSE; Avants et al., 2011) as well as the MATLAB implementation of the global structural similarity index (gSSim; Wang, Bovik, Sheikh, & Simoncelli, 2004). By evaluating different measures, insights about the registration quality from different perspectives were obtained. Besides the three global measures (MSE, MI, and gSSim), CC was included to gain information about the local registration accuracy. As stated earlier, the applied radiotracers were expected to predominantly accumulate in GM. The estimation of the metrics was limited to the voxels within the binary brain mask that was distributed in combination with the MNI 2009c version.

To analyze whether the different MNI spaces have any considerable impact on the resulting similarity metrics, the same approach as explained above was applied using the MNI linear T_1_ template as a target for the test subjects. Furthermore, we also tested whether the 8‐mm FWHM Gaussian smoothing applied to the [^15^O]H_2_O template significantly impacts the registration quality. This was done by filtering the constructed tracer‐specific templates using an 8‐mm FWHM Gaussian kernel and using these versions as PET targets. For this assessment, we also elected to use the MNI linear‐based reference files to rule out both effects.

## 4 RESULTS

### 4.1 Constructed atlases

**FIGURE 3:**
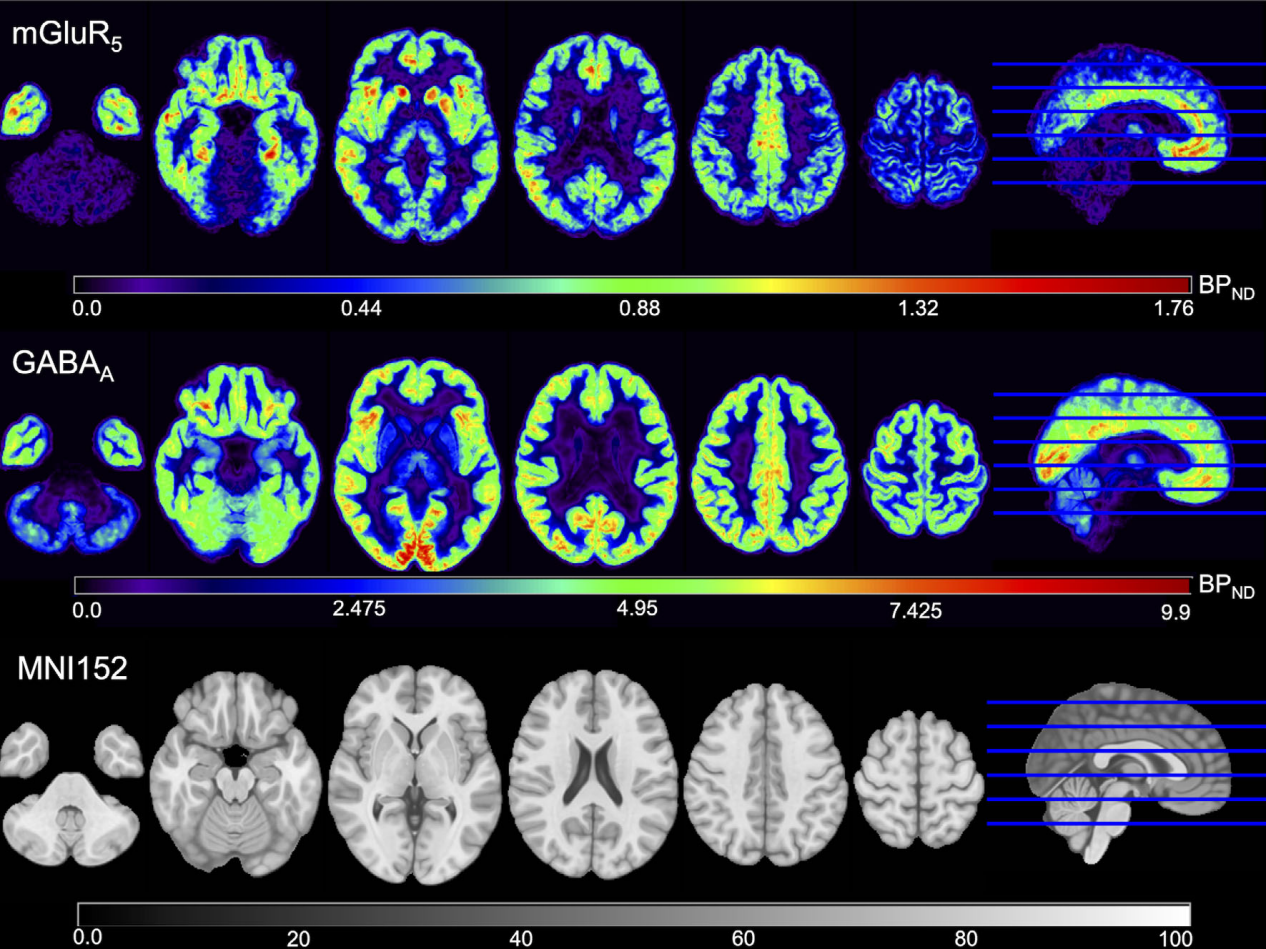
Example slices of the resulting mGluR_5_ (top) and GABA_A_ (middle) *BP*_ND_ atlases. The voxel‐wise *BP*_ND_ values are color‐coded. For anatomic reference, the corresponding slices of the MNI152 template used for registration are displayed on the bottom. The slices 40, 60, 80, 100, 120, and 140 are displayed

Example slices of the constructed *BP*_ND_ templates for GABA_A_ and mGluR_5_ are presented in Figure 3. The GABA_A_ template shows a considerably larger range of *BP*_ND_ values compared to the mGluR_5_ *BP*_ND_ template. *BP*_ND_ values for GABA_A_ follow a gradient from maximum intensity values in the occipital lobe to low values in the basal ganglia. Compared to the GABA_A_ availability distribution, the mGluR_5_ *BP*_ND_ values are less homogenous in the GM. Here, the highest values were observed in the basal ganglia, the insular cortex, and the anterior cingulate gyrus. The data for all regions in the Harvard‐Oxford cortical and subcortical atlas is given in the Supporting Information Material for all constructed maps.

Extensive data from other modalities for these two tracers were not readily available for a comprehensive comparison of the resulting data, so no validation was possible in this regard.

### 4.2 Assessment of the warp effect

**FIGURE 4:**
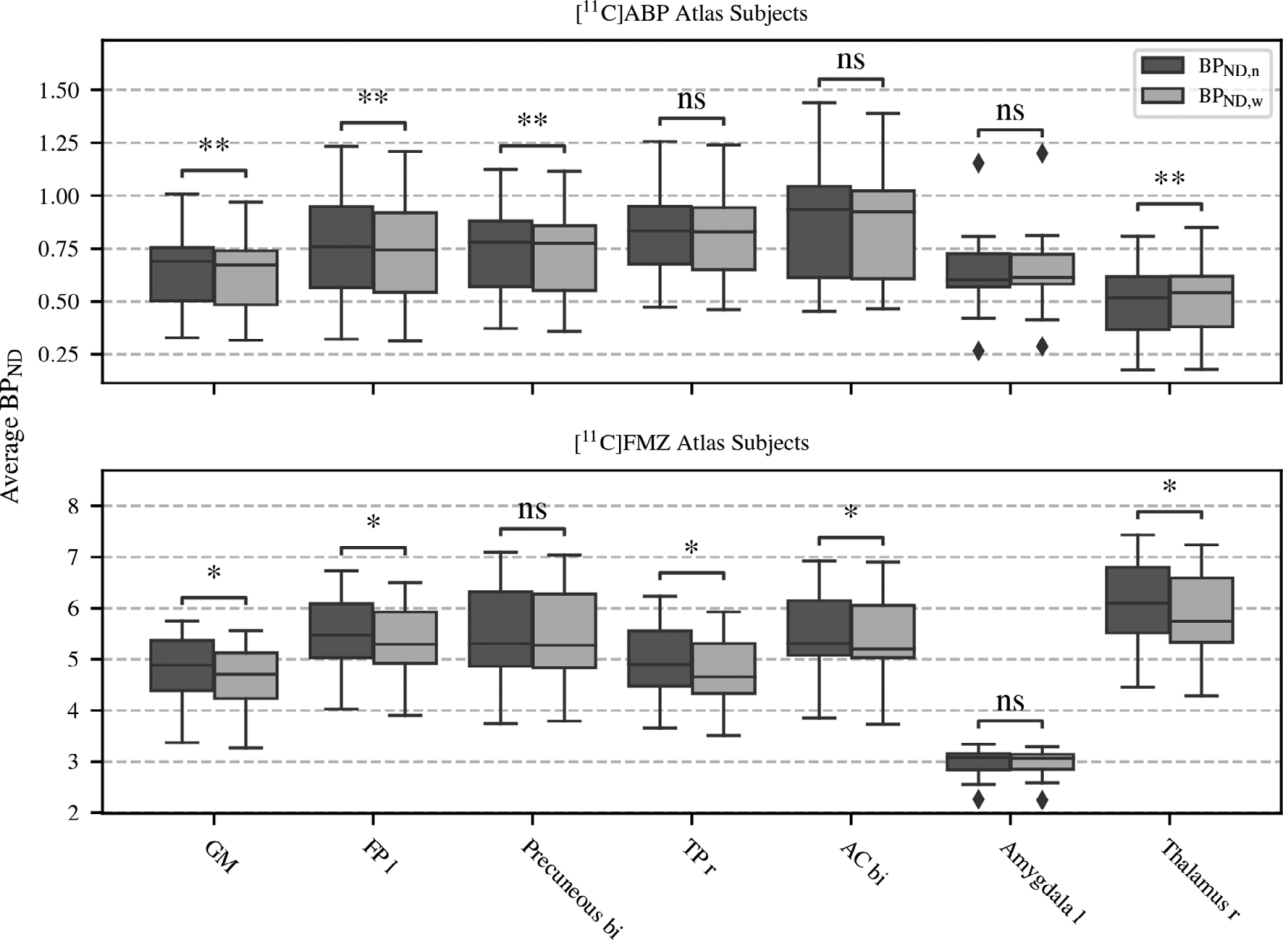
Boxplot graph comparing the average *BP*_ND_ values in native (*BP*_ND,*n*_) and warped space (*BP*_ND,*w*_) for the [^11^C]ABP subjects (top) and the [^11^C]FMZ subjects (bottom). The whiskers indicate 1.5 times the lower/upper quartile; values outside this range are marked as outliers. The significance of the difference between native and warped space according to the Wilcoxon signed‐rank test is indicated by asterisks. (*p<.05, **p<.01; AC, anterior cingulate; bi, bilateral region; FP, frontal pole; l, left; ns, not significant; r, right; TP, temporal pole)

The results for a selection of large, medium, and small ROIs for both sets of subjects are given in Figure 4. The assessment of the warp effect in general revealed only a very slight difference between the native and the warped space values for both subject groups. No clear systematic difference between the native space and warped space values was observed in either group. For the [^11^C]FMZ group, the maximal δ across all subjects in the selected ROIs was found in the GM at 7.8%. The highest average δ was observed in the right thalamus at (3.8 ± 1.4)%. The maximal δ in the [^11^C]ABP group was found in the right thalamus at 7.1%, and the left frontal pole exhibited the highest average δ of (3.0 ± 1.0)%. Despite the low differences, a two‐sided Wilcoxon signed‐rank test revealed that 9 out of 14 tested reasons were significantly different at a significance level of *p* < .05.

### 4.3 PET‐to‐PET registration tests

**FIGURE 5:**
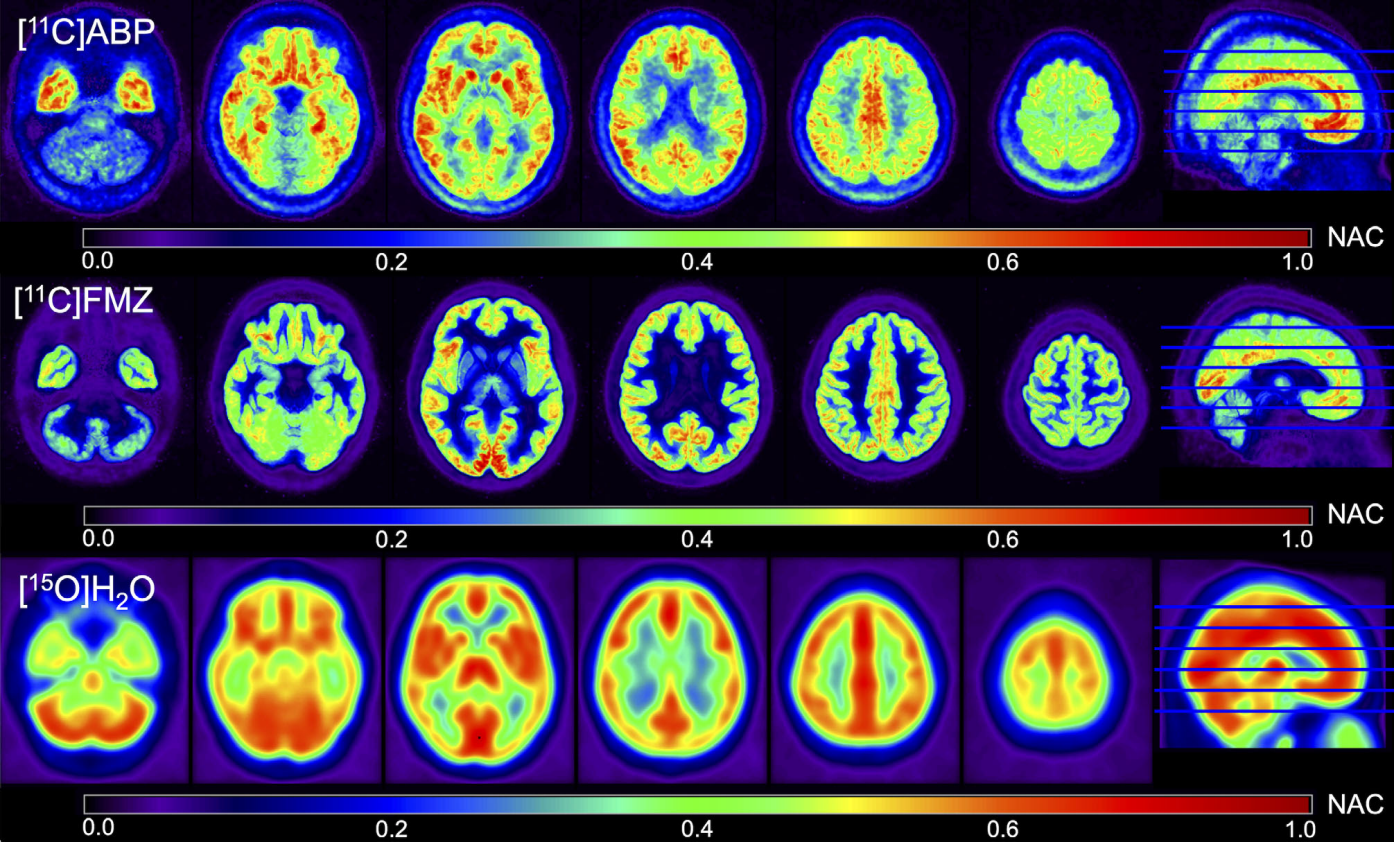
Example slices of the [^11^C]ABP (top) and [^11^C]FMZ (middle) normalized activity concentration templates in comparison to the corresponding slices of the [^15^O]H_2_O perfusion template (bottom). The activity concentration in each voxel is color‐coded according to the respective color bar and was normalized to 1 (NAC: normalized activity concentration) in all templates. The images are presented in radiologic orientation. The slices 40, 60, 80, 100, 120, and 140 are displayed

Example slices of the constructed normalized activity concentration templates of [^11^C]ABP and [^11^C]FMZ are illustrated in Figure 5. In order to demonstrate the more realistic tracer distribution of the tracer‐specific templates, the corresponding slices of the commonly used [^15^O]H_2_O template are also given. As expected, the activity concentration is predominantly distributed in GM for both tracers. The distribution in the [^11^C]ABP template is more homogeneously distributed across the whole GM in comparison to the [^11^C]FMZ data, where activity concentration hotspots are visible in the occipital lobe and the insular cortex. In comparison, the [^15^O]H_2_O template is very blurry due to the fact that an 8‐mm FWHM Gaussian kernel was used to smooth the files before generating the template. Furthermore, the [^15^O]H_2_O template shows almost no variation of activity within GM.

**FIGURE 6:**
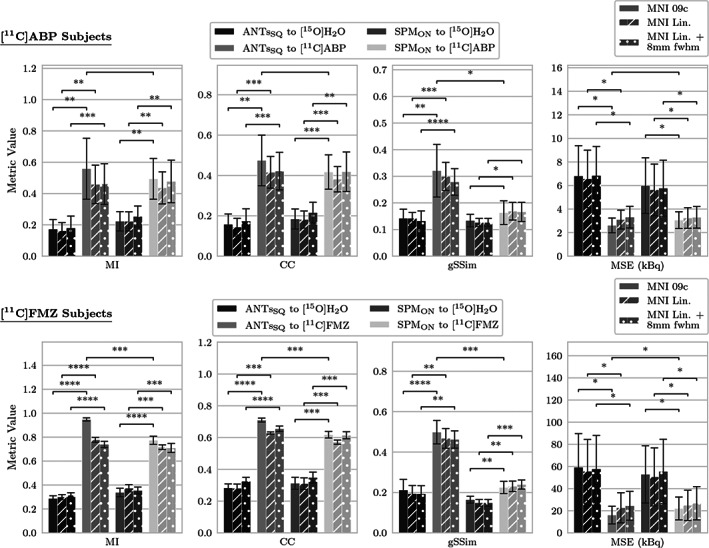
Evaluation of the registration quality using different templates and two registration methods: antsRegistrationSyNQuick (ANTs_SQ_) and SPM Old Normalize (SPM_ON_). Top: [^11^C]ABP test subjects were registered once to a perfusion [^15^O]H_2_O template and once to the specific [^11^C]ABP template. Bottom: The same was done with the [^11^C]FMZ test subjects. The evaluation was based on four kinds of similarity metrics (MI, mutual information; CC, local cross correlation; gSSim, structural similarity; MSE, mean squared error). The error bars indicate the standard deviation. Testing was performed once with the reference files being registered based on the MNI 2009c space (MNI 09c:plain pattern) and once towards the MNI linear space (MNI Lin., diagonal lines). Additionally, the new templates were smoothed to the same full‐width at half maximum (FWHM) as the [^15^O]H_2_O template (8‐mm FWHM) to ascertain whether it has any impact on the registration quality (dotted bars). Significance of the differences between the relevant groups is indicated by asterisks (*p<.05, **p<.01, ***p<.001, ****p<.0001) or not significant (no label), according to a paired two sample *t* test multiple comparisons correction was not applied

The results of the registration tests for both groups of subjects are given in Figure 6. Three different cases were evaluated: (a) using the MNI 2009c space template to register the reference images (indicated by the plain bars); (b) using the MNI linear space template for registration (diagonally lined bars); and (c) using the MNI linear space template as well as applying an 8‐mm FWHM Gaussian smoothing kernel to the tracer specific templates to match the amount of blur in the [^15^O]H_2_O template.

#### 4.3.1 General observations

The similarity metrics MI and CC are consistently higher for the registrations to the specific templates than the scores for the respective registrations to the [^15^O]H_2_O template for both the [^11^C]ABP and [^11^C]FMZ group. The MSE was visibly lower for both groups, but due to the high standard deviations in this metric, the differences are not as pronounced. gSSim differs from the other metrics, as the gSSim value seems to vary strongly with the applied registration method. The ANTs_SQ_ registrations to the tracer‐specific templates score considerably higher than the respective SPM_ON_ registrations. All average values and corresponding standard deviations are given in the Supporting Information Material. In addition to this qualitative evaluation, paired two sample *t* tests were applied to test the statistical significance of the differences in the evaluated metrics between the relevant groups. Testing was performed between tracer‐specific and arbitrary registration approaches, as well as for the registration routine used as the target template for the MR‐based reference images and smoothing of the new templates.

Except for the gSSim metric of the SPM_ON_ registration in the [^11^C]ABP group, all tested cases were found to be significantly different compared to the respective registration towards the [^15^O]H_2_O template.

The difference between the applied registration method was only significant in terms of gSSim in the [^11^C]ABP group, whereas it was significant in all tested cases in the [^11^C]FMZ subjects. The level of statistical significance of the difference in similarity metrics was generally higher in the [^11^C]FMZ group compared to the [^11^C]ABP subjects. This is a clear indication of the superior SNR in [^11^C]FMZ data.

Testing whether the difference in MNI spaces has a significant impact on the registration quality revealed that all cases which were found to be statistical significant in the previous case remained statistically significant in this test. Additionally, the difference in gSSim of the SPM_ON_ registration was found to be statistically significant in this case although it had been not statistically significant before.

Smoothing was shown to have almost no effect on the significance of any difference.

## 5 DISCUSSION

In this work, we aimed to construct parametric maps containing information about the normal *in vivo* neuroreceptor distribution of mGluR_5_ and GABA_A_ receptors in standard MNI space based on bolus‐infusion of [^11^C]ABP / [^11^C]FMZ PET and simultaneously acquired MR data. For this purpose, we assembled an innovative, easy to use, and comprehensive data pre‐processing pipeline. Following the revision and compilation of the processing steps, distribution maps of *V*_T_ and *BP*_ND_ of mGluR_5_ and GABA_A_ neuroreceptors were constructed. The main validation of the resulting maps focused on the assessment of the effect of the nonlinear transformation on the parametric image values. Our investigation revealed only a slight, yet, in many cases, significant, difference between the native and the warped space values for both neuroreceptor maps. Furthermore, we applied the pipeline to construct normalized activity concentration templates for direct PET‐to‐PET registrations, as no tracer‐specific templates for the two tracers used existed at the time of this investigation. The initial examination revealed that the PET‐to‐PET registrations gave higher accuracy when using the tracer‐specific PET templates compared to the commonly used [^15^O]H_2_O perfusion template. This is in agreement with previous investigations of other tracer‐specific templates (Della Rosa et al., 2014; Gispert et al., 2003; Meyer et al., 1999).

### 5.1 Considerations regarding pipeline construction

Our pipeline was assembled using the Python‐based neuroimaging package NiPype (Gorgolewski et al., 2011), which, due to its modular nature, offers the possibility of exchanging individual steps to adapt to specific needs. Furthermore, the proposed pipeline can be applied with minimal user intervention by simply providing the reconstructed bolus‐infusion PET data, raw structural MR scans, a target template file in NifTi format, and the material for *V*_T_ and/or *BP*_ND_ estimation as inputs. However, the metabolite corrected venous blood plasma activity at the time of interest must be given as an input to the pipeline in the form of a text file for estimation of *V*_T_, and a binary NifTi file masking the reference region is necessary as an additional input to calculate *BP*_ND_. Thus, the pipeline is not immediately applicable to all types of PET data in its current form.

A crucial step during the pipeline is the applied spatial normalization routine. There are two general approaches to spatial normalization of functional images which have been discussed previously (Ashburner & Friston, 1999): (a) direct calculation of the transformation parameters from the subject‐space PET image to a standardized PET template; (b) the use of a structural scan from another imaging modality, that is in co‐registration with the PET image, to estimate the transformation and then to apply this transformation to the PET image in a second step. Higher registration quality using the latter approach was first proposed by Ashburner and Friston (1999). Subsequently, this approach was investigated, and the findings of Gispert et al. (2003) and Martino et al. (2013) indicate a significantly improved registration quality when using an MR image to estimate the spatial transformation. This significant difference is due to the fact MR offers a superior spatial and structural resolution compared to PET (Ashburner & Friston, 1999), whereas PET is advantageous for the visualization of metabolic processes. Thus, most previous studies have employed varieties of an MR‐based approach to register PET images to a template space (Della Rosa et al., 2014; Gispert et al., 2003; Vállez Garcia et al., 2015). Due to the above advantages, we elected to apply the MR‐based method to estimate the transformation. However, the dynamic [^11^C]Raclopride template, described in Section 1 of this article, was constructed using a purely PET‐based approach (Bieth et al., 2013).

In the following, some methods applied in the proposed approach are briefly discussed. During this work, two bias correction methods were tested: the bias correction method included in the current segmentation routine of SPM12 and the N4 bias correction (Tustison et al., 2010) method that is included in ANTs. As realistic phantom data are difficult to obtain, the methods were assessed by visual inspection. Here, the N4 bias correction seemed to remove the bias field best when using the manually edited brain mask, restricting the algorithm to include only brain voxels in the estimation. The image intensity ranges were standardized to each other using an option in the ANTs N4 bias correction routine. To be able to perform *BP*_ND_ estimation, as well as being able to warp the parametric images, the corrected MR images were then warped into MNI 2009c nonlinear space. The ANTs registration routine was shown to outperform a variety of other methods in an evaluation of 14 nonlinear registration methods (Klein et al., 2009). In addition, ANTs offers several parameters that can be adjusted to optimize the output. Thus, ANTS registration was applied as the method of choice in this work.

An important quality control step is the examination of the resulting motion correction parameters. Observation of these parameters is necessary due to the attenuation correction method applied (see Section 2.1). Large movements cause errors in the reconstruction, and scans that contained motion larger than the image resolution were excluded from consideration. This was achieved by applying motion correction with respect to the first frame. Only scans with motion parameters less than 3 mm in *x*, *y*, *z*‐direction or 3.5° of roll, pitch, or yaw were considered for further processing.

### 5.2 Applicability of the tracer‐specific maps and templates

To be able to use the constructed *in vivo* maps of neuroreceptor distribution, it was necessary to assess the impact of the warp on the parametric values of the PET images. Therefore, the values in warped space were compared to native space values by calculating the percent error from the warped space values to native space values. To assess the resulting deviation, the test–retest variability of [^11^C]FMZ and [^11^C]ABP was taken into consideration. For [^11^C]FMZ, a percentage difference of 1.00%–6.36% was reported when using the more precise arterial input function for kinetic modeling and 3.89%–9.53% when applying modeling using the pons as reference region (Salmi et al., 2008). Thus, the highest average deviation observed in the sampled regions of 3.8 ± 1.4% lies well within the expected variability of [^11^C]FMZ. [^11^C]ABP has been shown to have a high intrinsic variability (DeLorenzo et al., 2011), especially for same‐day retests (DeLorenzo et al., 2017). A difference in *BP*_ND_ values of 4%–16% has been reported (Smart et al., 2018) when *BP*_ND_ was estimated using the simplified reference tissue model (Lammertsma & Hume, 1996). Therefore, the highest average deviation of 3.0 ± 1.0% is smaller than the expected general variability of [^11^C]ABP. Although the percent errors were lower than the expected variabilities of the tracers, a Wilcoxon signed‐rank test revealed that the differences were significant in 8 of the 14 tested cases. This suggests that the transformation, or the applied way of extracting the values, introduced a systematic bias towards lower values in warped space, which should be carefully considered before use.

We also applied the pipeline to generate MNI space activity concentration images that were normalized by the mean activity in GM. With these images, we constructed [^11^C]FMZ‐ and [^11^C]ABP‐specific normalized activity concentration templates for direct PET‐to‐PET registration. This kind of registration is necessary when no accompanying MR scan is acquired, and a more advanced analysis of the PET scan is needed. Only a very small number of MNI space PET templates are currently publicly available. If no tracer‐specific PET template is available, the [^15^O]H_2_O template distributed with SPM12 is usually used to estimate the transformation. However, as the [^15^O]H_2_O template was constructed based on an earlier version of the MNI152 space, its alignment with the most recent versions of the MNI space is poor. Small alignment deviations could lead to misestimations of parameters. Thus, the newly constructed tracer‐specific templates should be better suited for analyses with more recent brain parcellations. We evaluated the differences between the two template spaces as well as between the MR‐based and PET‐based registrations in several tests. The local and global differences between the [^15^O]H_2_O template distributed with SPM12 and the newly constructed templates are visualized in Figure 5. While, due to the perfusion‐weighted pattern, the distribution of activity concentration in the [^15^O]H_2_O template mostly resembles GM, the [^11^C]ABP and [^11^C]FMZ templates exhibit a much more differentiated distribution according to their respective tracer characteristics. Furthermore, the [^15^O]H_2_O template appears blurry compared to the [^11^C]ABP and [^11^C]FMZ templates. This is because an 8‐mm FWHM Gaussian kernel was used to smooth the data before constructing the template. Based on this, we hypothesized that registration algorithms will likely be able to match finer details of a PET image using the specific templates. This assumption was subsequently investigated with a quantitative analysis of several image similarity metrics. Native PET images of the test subjects were transformed into MNI space using the [^15^O]H_2_O and the respective tracer‐specific templates as targets. To assess whether the applied registration routine influenced the results, all registrations were estimated twice using antsRegistrationSyNQuick and the SPM12 routine Old Normalize. The resulting images were then compared to the MR‐based and partial‐volume corrected transformation of the same file using the image similarity metrics MI, CC, gSSim, and MSE. A paired two‐sample *t* test revealed significant differences in all applied tests of the registration accuracy of tracer‐specific registrations in comparison to a perfusion‐based registration. The only exceptions to this were two cases, both in the gSSim metric of the SPM_ON_ routine in the [^11^C]ABP group. This is likely an indication of the superior registration accuracy of ANTs_SQ_ when compared to SPM_ON_ which is in accordance with the findings of (Klein et al., 2009). Furthermore, it could mean that gSSim is more sensitive to registration accuracy in comparison to other similarity metrics.

Due to the lower accuracy and the misalignment of this target in comparison to the MNI 2009c, the similarity metrics would be expected to be significantly lower if the misalignment of the MNI spaces plays any role in terms of similarity. Additionally, the [^15^O]H_2_O was heavily smoothed due to the high positron range of ^15^O (Herzog, 2018). To ensure that this lower resolution is not the main cause of the lower registration quality, we applied the same kernel to the novel [^11^C]ABP and [^11^C]FMZ templates and repeated the tests. In both cases, only the level of statistical significance changed in some cases, but all tests remained statistically significant.

### 5.3 Limitations

When interpreting our study, some limitations should be kept in mind. First, although fully automatic processing is an implemented option in the pipeline, it is questionable whether current skull stripping methods can provide the necessary accuracy in a robust way. Therefore, we chose to manually edit brain masks in a prior step and give them as input into the pipeline in addition to the raw PET and MR images. A robust skull‐stripping routine that offered results visually imperceptible from manual extractions would improve the usability of the proposed pipeline considerably.

Second, the quality of the intermediate steps that is, motion correction, co‐registration, PVC, and nonlinear registration, ultimately limits the quality of the resulting images but is often impossible to quantify. Thus, these steps were visually inspected for inconsistencies, which is an error‐prone inspection method.

Third, the considered datasets comprise a relatively small sample size. Thus, the conclusions drawn need to be considered with care and can only be seen as indications of the truth. Extensions of the databases would greatly improve the robustness of the resulting templates. Furthermore, the pons was used as a reference region with no specific binding for the estimation of *BP*_ND_ for the [^11^C]FMZ data in this work. This topic is controversially discussed in the literature, as a previous study has revealed that the pons is not entirely devoid of GABA_A_ receptors (Delforge et al., 1995). The use of the pons as the reference region for studies with [^11^C]FMZ was investigated extensively in Klumpers et al. (2008), where they concluded that quantification of binding using the pons as reference region is feasible for most clinical purposes. Nonetheless, a slight underestimation of the true *BP*_ND_ in the [^11^C]FMZ results should be expected.

Furthermore, no extensive comparison of the results with existing gene expression or histology data was performed to further validate the results. This could be achieved by performing analyses on the basis of the Allen Human Brain Atlas (Hawrylycz et al., 2012) as well as the Jülich Histological Atlas (Eickhoff et al., 2007, 2005; Eickhoff, Heim, Zilles, & Amunts, 2006). However, these extensive analyses would significantly extend the scope of this work, and the results obtained do not indicate a need for additional validation. Furthermore, the *in vivo* data of our atlases might not entirely correlate with the *ex vivo* data of gene expression and histology data. After the major measurements and analyses for this work had already been completed, another group of researchers published results of a high resolution GABA_A_ atlas (Nørgaard et al., 2021) in which gene expression and histology were considered. It may thus be possible to further validate our results using the aforementioned tools in an ensuing study.

Finally, in addition to the very low sample size (four and five subjects respectively), the approach applied to evaluate the registration quality is intrinsically limited. The tests were based on the assumption that MR‐based registrations of PET images are more accurate than intermodal registrations of PET images, as previously hypothesized in Ashburner and Friston (1999) and reviewed in Gispert et al. (2003) and Martino et al. (2013). However, MR‐based registrations cannot be expected to be without errors, meaning that the “ground truth” images to which the results were compared do not actually reflect a ground truth. This test could be improved by using simulated data to quantify the registration accuracy in a more robust way.

## 6 CONCLUSION

In this work, we demonstrate the reliable applicability of a PET/MR NiPype image processing pipeline specifically designed to give accurate, parametric *in vivo* PET template construction. The pipeline provides a neuroimage pre‐processing method that is easy to implement for arbitrarily large bolus‐infusion PET/MR datasets using state‐of‐the‐art image processing tools at minimal user intervention. The pipeline outputs were further used to construct MNI space distributions of *V*_T_, *BP*_ND_, and normalized activity concentration of [^11^C]FMZ and [^11^C]ABP in healthy male humans. The validations performed lead to the conclusion that the pipeline is suitable for application in a variety of advanced analyses of multimodal datasets. However, the constructed maps should only be used after careful consideration of the warp effect. Based on the extensive testing of the applicability of the normalized activity concentration templates for PET to PET registration, we conclude that using the constructed templates instead of an arbitrary perfusion template should benefit registration performance.

## CONFLICT OF INTERESTS

The authors declare that there are no conflict of interests.

## AUTHOR CONTRIBUTIONS

**Nicolas Kaulen**: Conception of PET/MR processing pipeline and other methodology, evaluation of processing methods, data acquisition, metabolite correction, and paper writing and correction. **Ravichandran Rajkumar**: Study and experimental design, supervision of the project, methodological considerations, paper writing, data acquisition, and revision of the paper. **Claudia Régio Brambilla**: PET expertise, metabolite correction, bolus‐infusion optimization, data acquisition, correction, methodological considerations, and revision. **Jörg Mauler**: PET bolus‐infusion study design, data acquisition, and revision of the paper. **Shukti Ramkiran**: Data acquisition, methodological considerations, and revision of the paper. **Linda Orth**: Volunteer screening and data acquisition. **Hasan Sbaihat**: Data acquisition, methodological considerations, metabolite correction, and revision of the paper. **Markus Lang**: Radiotracer production and quality control. **Christine Wyss**: Study design and revision of the paper. **Elena Rota Kops**: MR‐PET attenuation correction, revision, and correction of the paper. **Jürgen Scheins**: PET reconstruction and revision of the paper. **Bernd Neumaier**: Radiotracer production supervision, revision of the paper. **Johannes Ermert**: Expertise in radiochemistry, revision, and correction of the paper. **Hans Herzog**: MR/PET maps discussions, methodological considerations, revision, and corrections of the paper. **Karl‐Joseph Langen**: Simultaneous MR‐PET acquisition protocol, revision of the paper. **Christoph Lerche**: Simultaneous MR‐PET acquisition protocol, PET data analysis pipeline, revision of the paper. **N. Jon Shah**: MR‐PET hardware and revision of the paper. **Tanja Veselinović**: Methodological considerations, paper writing, and correction and revision. **Irene Neuner**: Study design and setup, approval ethics and BfS, funding, and revision of the paper.

## ETHICS STATEMENT

Both studies of which data were analyzed in this work, as well as the corresponding analyses, were approved by the Ethics Committee of the Medical Faculty at the RWTH Aachen University and the German Federal Office for Radiation Protection (Bundesamt für Strahlenschutz). Written informed consent was obtained from all participants before the measurement.

## Supporting information


**Data S1:** Supporting Information: Time‐activity curves for the considered [^11^C]FMZ subjects.

**Data S2:** Supporting Information: Time‐activity curves for the considered [^11^C]FMZ subjects.

**Data S3:** Supporting Information: Example *V*_T_ data corresponding to the warp effect BP_ND_ data that was used for the evaluation of the warp effect.

**Data S4:** Supporting Information: Detailed BP_ND_ and *V*_T_values in the Harvard‐Oxford Atlas brain regions extracted from the mGluR_5_ and GABA_A_ PET distributions

**Data S5:** Supporting Information: A detailed NiPype‐generated graph of the applied processing steps in the pipeline

## Data Availability

The data that support the findings of this study as well as the resulting maps and templates are openly available in Jülich DATA at https://doi.org/10.26165/JUELICH-DATA/HDVEEF.
