# Supplementary figures and images for: mGluR5 and GABAA receptor‐specific parametric PET atlas construction—PET/MR data processing pipeline, validation, and application
Source: Hum Brain Mapp. 2022 Jan 25;43(7):2148–63. doi: 10.1002/hbm.25778 (PMC8996359; doi:10.1002/hbm.25778)

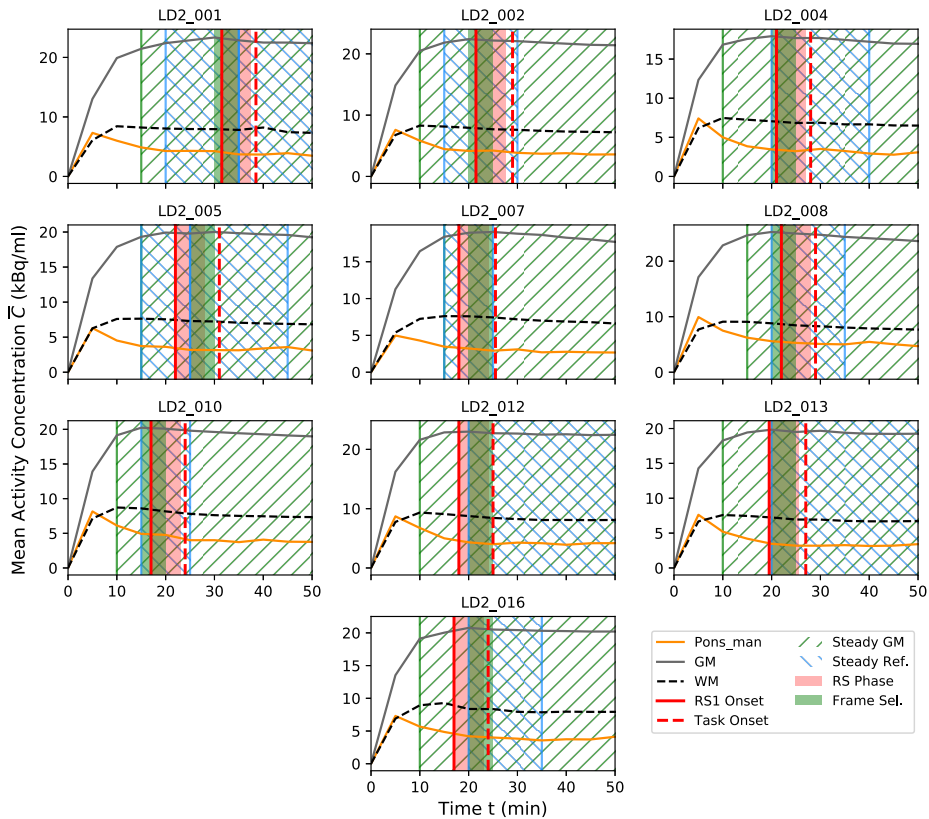

Supplement: Supplementary file 1 — Data S1: Supporting Information: Time‐activity curves for the considered [11C]FMZ subjects. [file HBM-43-2148-s001.pdf]

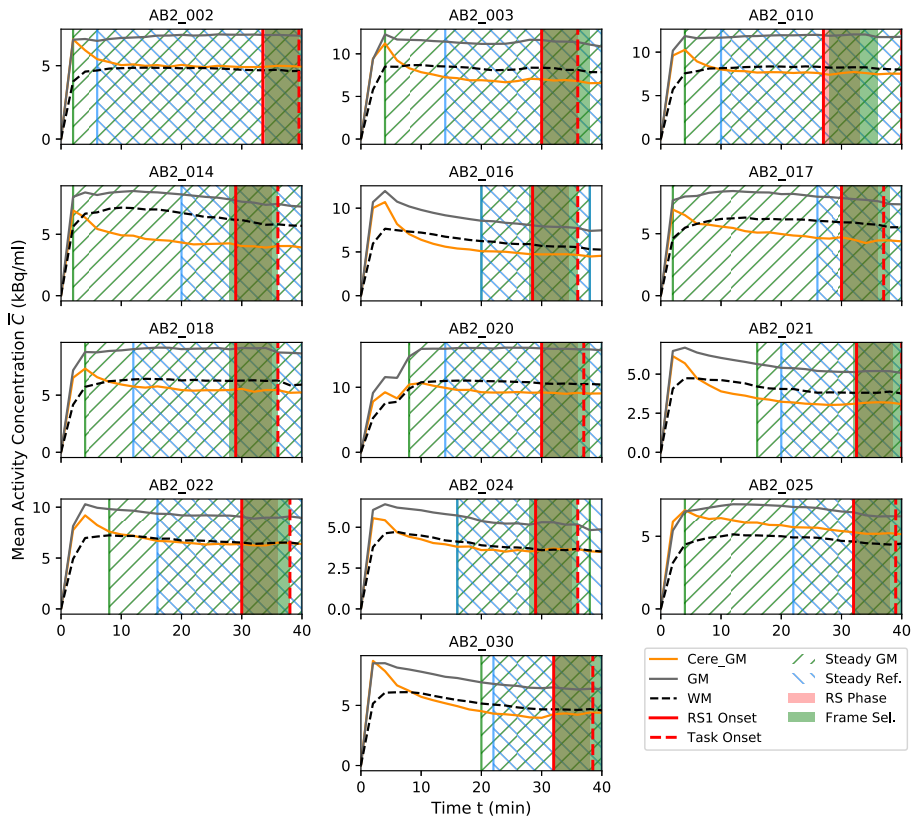

Supplement: Supplementary file 2 — Data S2: Supporting Information: Time‐activity curves for the considered [11C]FMZ subjects. [file HBM-43-2148-s004.pdf]

[<sup>11</sup>C]ABP Atlas Subjects

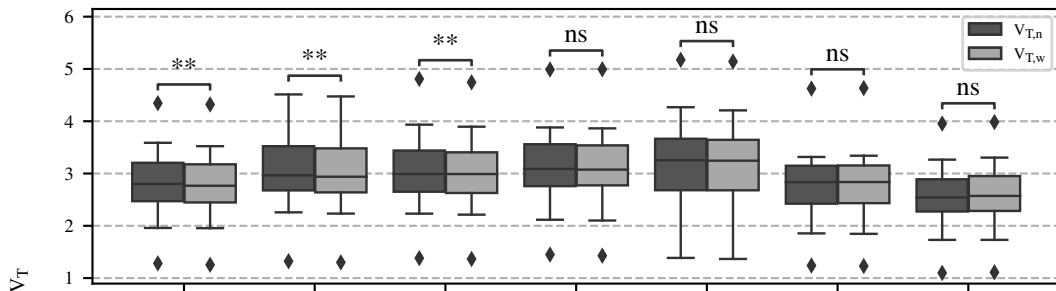

[<sup>11</sup>C]FMZ Atlas Subjects

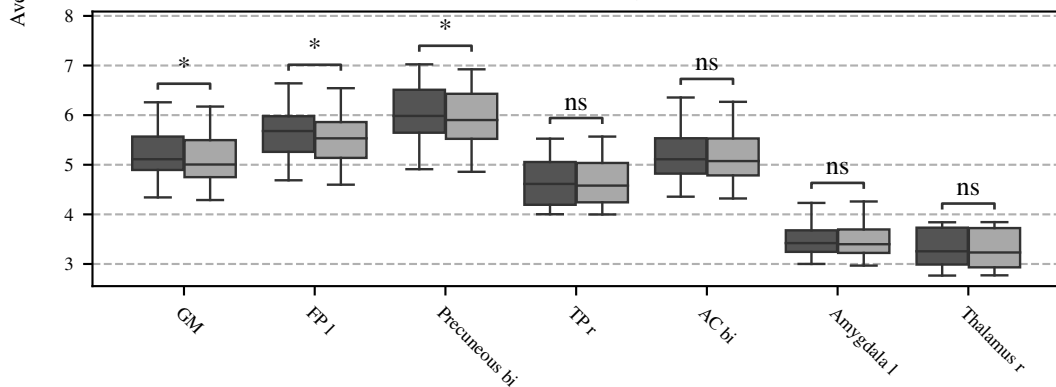

Supplement: Supplementary file 3 — Data S3: Supporting Information: Example V T data corresponding to the warp effect BPND data that was used for the evaluation of the warp effect. [file HBM-43-2148-s002.pdf]

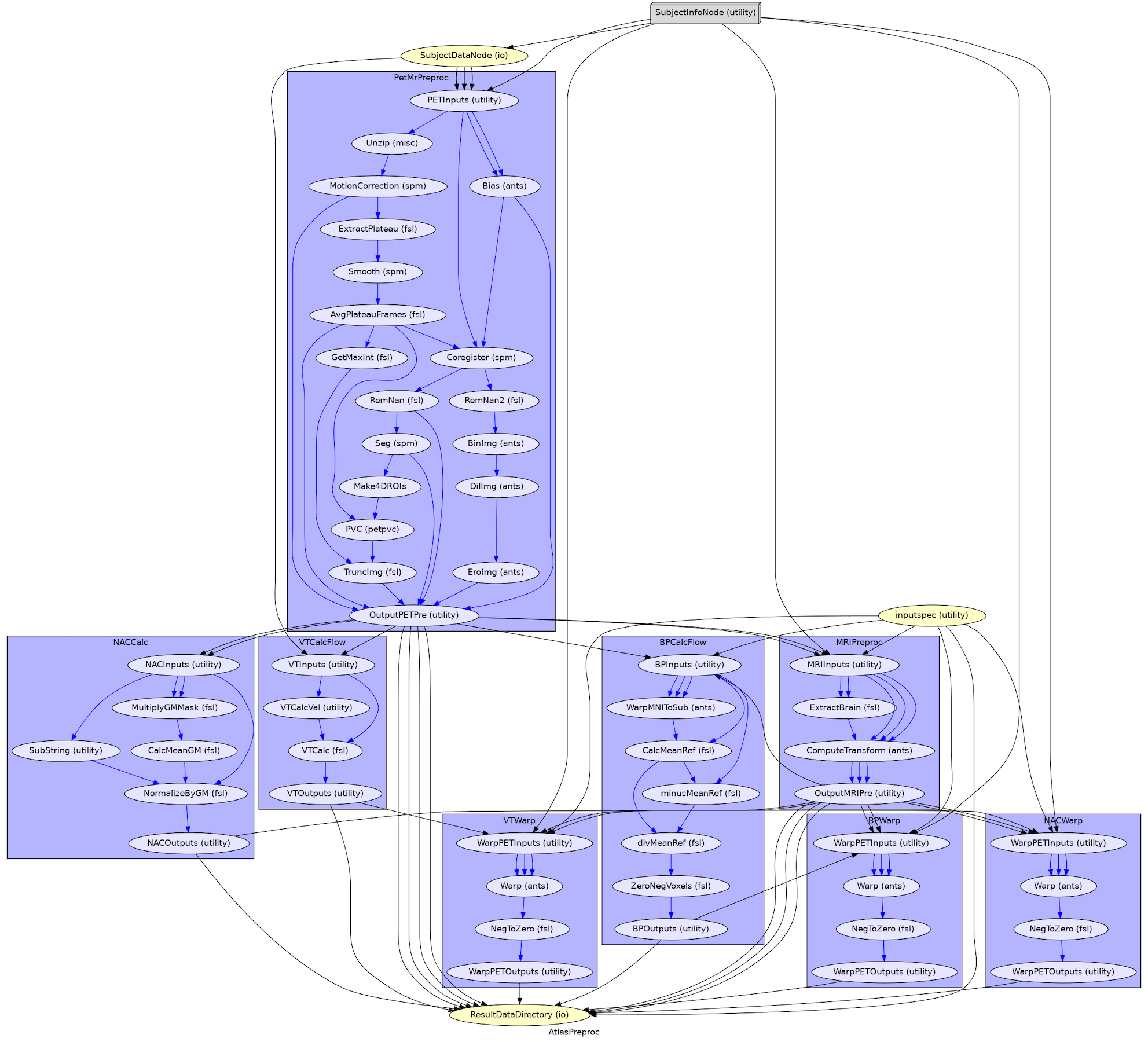

Supplement: Supplementary file 5 — Data S5: Supporting Information: A detailed NiPype‐generated graph of the applied processing steps in the pipeline [file HBM-43-2148-s005.png]
